# Being the Pillar for Children with Rare Diseases—A Systematic Review on Parental Quality of Life

**DOI:** 10.3390/ijerph18094993

**Published:** 2021-05-08

**Authors:** Johannes Boettcher, Michael Boettcher, Silke Wiegand-Grefe, Holger Zapf

**Affiliations:** 1 Department of Child and Adolescent Psychiatry, Psychosomatics and Psychotherapy, University Medical Center Hamburg-Eppendorf, 20251 Hamburg, Germany; swiegand-grefe@uke.de (S.W.-G.); h.zapf@uke.de (H.Z.); 2 Department of Pediatric Surgery, University Medical Center Hamburg-Eppendorf, 20251 Hamburg, Germany; m.boettcher@uke.de

**Keywords:** quality of life, parents, rare diseases, systematic review, caregivers

## Abstract

Parents caring for children with rare diseases fear the long-term progression of the child’s disease. The current study aims to systematically investigate the quality of life (QoL) in parents of children with different rare diseases. We performed a systematic literature search including quantitative studies on QoL of parents caring for children and adolescents with rare diseases in five databases (APA PsycArticles, APA PsycInfo, MEDLINE, PSYNDEXplus, and PubMed) published between 2000–2020. Of the 3985 titles identified, 31 studies met the inclusion criteria and were selected for narrative review. Studies were included if they investigated predictors of parental QoL or reported QoL compared to normative samples, parents of healthy children, or children with other chronic diseases. We used the Newcastle–Ottawa Scale to assess methodological quality. The systematic review revealed that parents of children with rare diseases experience reduced QoL compared to parents with healthy children and norm values. Psychosocial factors, beyond disease-specific predictors, were shown to influence parental QoL substantially and may thus present an essential aspect within interventions for this highly burdened group. Health care professionals should consider and address the impairment of parental QoL due to the child’s rare disease. We discuss insights into existing research gaps and improvements for subsequent work.

## 1. Introduction

Parents of children with rare diseases play a crucial role in the physical and emotional well-being of their affected child [1]. However, the difficulties faced by these parents throughout their lives are manifold including high parental responsibilities due to disease-specific requirements, limited access to adequate, quality health care, and lack of experienced health care professionals to provide appropriate treatment [2].

In the European Union, around 13.5 to 25 million children and adolescents are affected by one of over 5000–8000 different rare diseases [3]. Rare diseases are defined by a prevalence of less than 1:2000 [4]. Even though there is great heterogeneity in the complex disease patterns of rare diseases, the burden on the affected patients and their families is very similar [5,6]. The similar burden may stem from rare diseases mostly being severe, chronic, progressive, degenerative, and associated with a shortened life expectancy [2]. Moreover, it is assumed that about 80% of rare diseases are genetically caused or have genetic risk factors [7]. Furthermore, rare diseases are often detected late, and treatment options are limited, adding to the high level of pain and suffering endured by patients and their families [8,9].

The associated burden for the caring families, especially for the parents themselves, is often detrimental [5,10]. Challenges faced by parents caring for children with rare diseases can be manifold and include impairments regarding economic, psychosocial, and physical well-being [11]. Moreover, these diseases have a substantial impact on the parents’ lives, who may perceive an impairment in their professional, social, and family life, leading to a decreased quality of life (QoL). QoL can be defined as an “individual’s perception of their position in life in the context of the culture and value systems in which they live and in relation to their goals, expectations, standards, and concerns” [12]. The multidimensional concept of QoL comprises all substantial aspects of the life of each individual. To understand the psychosocial mechanisms that influence parental QoL, theoretical frameworks like the Caregiving Process and Caregiver Burden Model are substantial [13]. In this framework, the association between caregiving and psychological health is generally determined regarding the direct and indirect effects of the children’s characteristics, caregiver stress, and supportive factors (coping, social support, and family functioning) [14]. Theoretical frameworks containing stress and coping seem to be especially useful for parental QoL research [15].

Recent studies on the parental psychosocial effects of caring for children with rare diseases have focused on qualitative rather than quantitative approaches [5,16]. Although there is previous quantitative research on QoL of patients with rare diseases [17] and their siblings [18], to date, no systematic review on the quantitative literature has been published on the QoL of parents caring for children with rare diseases. QoL measures may provide a more comprehensive assessment of parental well-being [19]. Moreover, the assessment of QoL may help identify specific subgroups of diseases at risk of impaired function as well as protective factors that mitigate the adverse effects of caring for a rare diseased child [20].

This research aims to inform and contribute to improved psychosocial care for parents and their families. QoL is an important factor in understanding how parents respond and cope with the challenges of the child’s rare disease and other stressors. Information about the parents’ QoL can thus provide targets for future interventions. We conducted a systematic review of the available quantitative research to address the lack of compiled knowledge regarding the QoL of parents caring for a child with a rare disease. Our goal was to provide an overview of the parents’ shared psychological experiences affected by their children’s rare diseases without solely focusing on a specific disease group. Three key questions provide a focus for this systematic review: (1) How does QoL in parents of children with rare disease compare to QoL in parents of healthy children, general population norms, or parents of children with chronic diseases? (2) What are the psychosocial and disease-specific predictors of QoL as a primary outcome of parents caring for a child with a rare disease? (3) Are the findings regarding QoL in parents of children with the same rare diseases consistent?

## 2. Materials and Methods

We conducted this systematic review according to the Preferred Reporting Items for Systematic Reviews and Meta-Analyses (PRISMA) guidelines [21]. Supplementary File S1 shows the corresponding PRISMA 2009 checklist from Moher et al., 2009 [21]. We searched the PROSPERO database [22] to ensure that no similar studies have been started or planned and published a protocol for this study under the number CRD42020187929.

### 2.1. Search Strategy

We conducted the search and selection process between March 2020 and November 2020, identifying original studies by searching five electronic databases including APA PsycArticles (Ovid), APA PsycInfo (Ovid), MEDLINE (Ovid), PSYNDEXplus (Ovid), and PubMed. The references of all selected publications were searched for additional studies. Table 1 presents the search strategies used via Ovid databases and PubMed.

### 2.2. Eligibility Criteria

The eligibility criteria were determined using the PICO characteristics [23] (i.e., characteristics describing the study population (P), intervention/exposure (I), comparison condition (C), outcome (O), and study design (S)). All original, peer-reviewed articles published in English or German, addressing the QoL (O) of parents (P) caring for a child with a rare disease (I) according to the definition of the European Commission (<1:2000), based on quantitative methodology using standardized, validated questionnaires, and published from 2000 to 2020 were included. A 20-year time period was chosen because studies within this period are more likely to reflect current health care policies in the respective countries [24]. The comparison condition characteristic (C) was defined as parents of healthy children, general population norms, or parents of children with chronic diseases. We only included papers on multiple disease groups if the results were presented separately for the specific rare disease groups. The following studies were excluded: case studies, unpublished dissertations, clinical trials of drug, surgery, psychosocial or medical interventions, validation studies of QoL instruments, qualitative studies, and studies on children’s experiences (S).

### 2.3. Data Extraction and Synthesis

**Figure 1:**
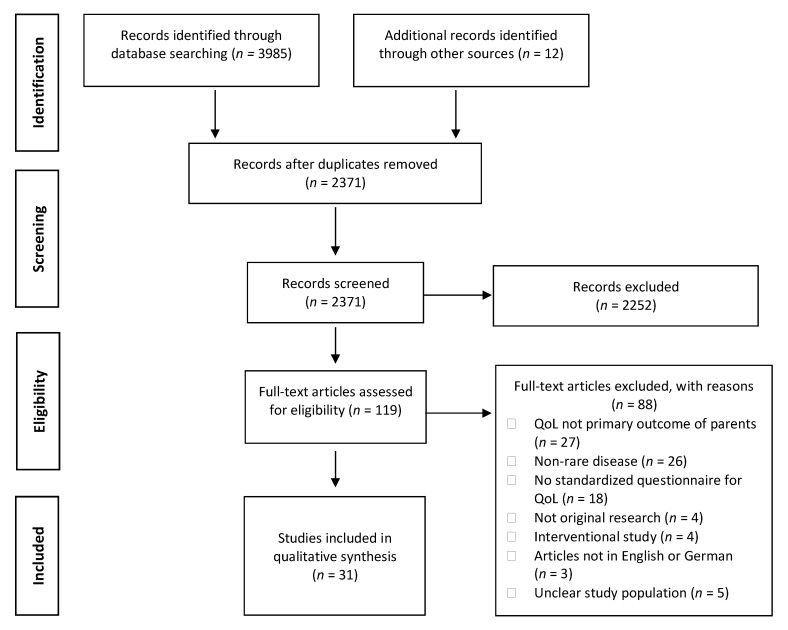
PRISMA flow diagram for the literature search and selection process. Source: Own elaboration based on the data obtained in the study.

We used the Mendeley Reference Manager to merge all search results and remove duplicates. The authors J.B. and H.Z. independently screened the titles and abstracts of studies retrieved using the search strategy and those from additional sources to identify studies potentially eligible for inclusion. It was decided whether the studies met the inclusion criteria. J.B. and H.Z. solved any disagreement through discussion. If necessary, the author S.W.G. was consulted. Multiple reports of a study were treated as a single study. Figure 1 shows the PRISMA flow diagram of the identified and selected articles [21]. Two authors (J.B. and H.Z.) independently conducted the data extraction. Information extracted included (1) study characteristics (first author, year, and country of publication, study design, sample size); (2) patient and adult caregiver characteristics (type of rare disease, gender of parents, age range of children); (3) type of QoL instruments as well as (4) selected findings (statistical comparisons with healthy controls, population norms, or other disease populations, analysis of associations with important disease-specific, and psychosocial predictors). Summary and evidence tables were created for this purpose. Since the studies were too heterogeneous, we performed a narrative synthesis of the results.

### 2.4. Assessment of Methodological Quality

The authors J.B. and H.Z. independently assessed the methodological quality and risk of bias of the studies using a modified Newcastle–Ottawa Scale for cross-sectional studies [25] and the Newcastle–Ottawa Scale for cohort studies [26]. These scales score studies based on three categories (selection, comparability of study groups, and outcome of interest). Cross-sectional studies could score a maximum of four points regarding selection, two points for comparability, and two points for outcome. Based on the overall score of each study, the quality was categorized as: very good (7–8 points), good (5–6 points), satisfactory (4 points), or unsatisfactory (0–3 points), according to the classification adopted by Ogden et al., 2020 [27]. Cohort studies could score a maximum of four points regarding selection, two points for comparability, and three points for outcome. We used the criteria adopted by Donzelli et al., 2019 [28] to classify the overall assessment of study quality as follows: very good (8–9 points), good (6–7 points), satisfactory (4–5 points), and unsatisfactory (0–3 points). Disagreements in scores regarding the quality assessment were resolved by discussion and consensus.

## 3. Results

### 3.1. Study Selection

In the literature search, we identified 2371 articles after removing duplicates. Inter-rater reliability reached moderate agreement between the two officers’ judgment *k* = 0.542 (95% CI, 0.448 to 0.635), *p* < 0.001. We selected 119 articles for full-text analysis, resulting in the exclusion of 88 articles. Subsequently, 31 articles met all inclusion criteria and were included in the systematic review. The PRISMA flowchart is presented in Figure 1.

### 3.2. Methodological Quality of Included Studies

Supplementary Files S2 and S3 illustrate the quality of the included studies using the Newcastle–Ottawa Scale for cross-sectional studies and cohort studies. Of the cross-sectional studies, ten studies (35.7%) were assessed as very good quality, nine as good quality (32.1%), and nine as satisfactory quality (32.1%). In contrast, of the cohort studies, two studies were assessed as good quality (66.7%) and one study as satisfactory quality (33.3%). No studies were of unsatisfactory quality.

### 3.3. Study Characteristics

The study characteristics of the 31 included studies are summarized in Supplementary File S4. The included studies stemmed from 10 different countries: Australia [29], Brazil [30], Canada [31], Germany [32,33,34,35], Iran [36,37,38], Ireland [39], the Netherlands [40,41,42,43,44,45], Poland [46], Sweden [47], and the U.S. [48,49,50,51,52,53,54,55,56,57,58,59]. Of the 31 studies, 28 used a cross-sectional design [29,30,31,32,33,34,35,36,37,38,39,40,41,42,43,44,46,47,48,50,51,52,53,54,55,56,58,59], whereas three were cohort studies [45,49,55]. Table 2 lists the children’s rare diseases by clinical category, which included chromosomal disorders [40,53,54,58], congenital diseases [34,44,45], hematologic and oncologic diseases [33,37,43,47,56,58], inflammatory diseases [39,41,48,49,59], metabolic disorders [32,36,38,42,50,51,57], musculoskeletal diseases [30,35,46,52], and neuromuscular and neurologic disorders [29,31,40,55]. Four studies examined only mothers [37,43,54,55], 27 studies examined both mothers and fathers [29,30,31,32,33,34,35,36,38,39,40,41,42,44,45,46,47,48,49,50,51,52,53,54,55,58,59]. Five studies did not differentiate between male and female caregivers [31,36,50,51,58]. The gender distribution showed a higher percentage of women compared to men in all studies. There was a large variance in sample size within the included studies ranging from 12 to 326 caregivers. Of all studies, 26 included more than 30 participants, while 11 studies even included more than 100 participants. Only three studies reported sample size estimations and power analyses [37,51,52].

### 3.4. QoL Instruments

Table 3 lists the different QoL instruments used in the studies. In this regard, a variety of QoL instruments was used. The most frequently used instrument was the PedsQL™ Family Impact Module, used in seven studies [31,50,52,53,54,55,56]. A wide range of studies also used the Short Form Inventory in the different versions SF-8 [34,35], SF-12 [29,49,56], and SF-36 [36,47,55]. Three different disease-specific QoL instruments, the CQOLC for cancer [37], the CQOLCF for cystic fibrosis [48,49,59], and the TYR-QOL for tyrosinemia [59], were used. A considerable proportion of 11 studies specifically used Health-Related Quality of Life (HRQoL) rather than QoL instruments [31,40,41,42,43,50,52,53,54,55,56]. Although the different instruments have different subscales, a distinction can be made in principle between the broader QoL categories of physical and psychosocial components.

### 3.5. Results from the Comparative Studies

A summary of the results of each study can be found in Supplementary File S4. A large proportion of the studies found that parental QoL was significantly lower than QoL of parents of healthy controls [42,43] or norm values [29,30,34,35,40,41,43,47,56,58]. There was a distinct pattern regarding the difference between psychosocial and physical QoL: Most studies found no impairment in the physical subscale, but significantly lower QoL compared to norm data in the psychosocial subscales [29,34,35,40,41,47,58]. However, two studies found a significant impairment in both physical and psychosocial QoL compared to norm data [30,43]. In one study, parents of children with a rare disease even showed higher physical QoL compared to U.S. female norms [29]. Another study found no significant difference between the QoL of parents of children with a rare disease and norm values of parents of healthy children [46]. When compared to parents of healthy children, parents caring for children with a rare disease experienced significantly lower QoL [43], whereas, in another study, parents of children with a rare disease perceived a comparable QoL to parents of healthy children and a significantly better QoL than parents of children with other chronic diseases [42].

Three studies investigated the differences in QoL between parents of children with rare diseases and those with other chronic diseases [31,33,57]. Campbell et al. (2018) found that parents of children with tyrosinemia type 1 had higher QoL than parents with mild phenylketonuria [59]. Wiedebusch et al., 2008, on the other hand, found that parents of children with hemophilia had significantly higher QoL compared to parents of children with juvenile idiopathic arthritis and type I diabetes [33]. In contrast, O’Mahony (2019) found significantly lower QoL in parents of children with pediatric multiple sclerosis than parents of children with monophasic acquired demyelinating syndrome [31]. Three studies found that parents of children with rare diseases showed significantly lower QoL in comparison to the norm data of parents of children with chronic health conditions living in a long-term care facility, whereas no significant difference was found for parents of children with a chronic condition living at home [50,51,54].

Five studies investigated gender-specific effects on parental QoL [32,33,34,42,44]. While one study found mothers to have a lower psychosocial QoL than fathers [34], other studies found no significant gender differences [32,33,42,44].

### 3.6. Predictors of Parental QoL

#### 3.6.1. Disease-Specific Predictors

Of the 31 included studies, seven studies investigated an association of QoL with disease-specific factors [30,39,48,51,52,55,59]. Among these factors are disease severity [48,51,55,59], child’s physical functioning and pain [30,54], hospitalization [48], and infection [39]. As expected, disease-specific factors were negatively associated with parental QoL. This is in accordance with some studies that investigated subgroups within the respective diseases [41,46,47,50,53,56]. These studies found that parents of children with a more severe form of the respective disease tended to have a lower QoL than parents of children with a milder form. However, a study conducted by O’Mahony (2019) found that chronicity, and not the severity of the disease, accounts for parental QoL [31]. Interestingly, one study found that disease severity was associated with poorer physical QoL, but improved psychosocial QoL [57]. Moreover, two studies found that as the child’s hospitalization days [48] and the time on enzyme replacement therapy increased, the caregiver QoL increased as well [53]. In another case, disease severity in terms of cardiovascular defects was associated positively with a significantly higher physical and emotional QoL in affected caregivers than caregivers of children with less severe defects in the same sample [55].

#### 3.6.2. Psychosocial Predictors

Thirteen out of 31 studies investigated associations between QoL and psychosocial predictors [29,30,32,33,34,35,36,37,38,39,42,51,57]. Such psychological factors of lowered parental QoL included elevated stress [32,36,38], elevated depressive and anxious symptoms [38,51], and lowered social support [32,42]. Identified social factors for lowered parental QoL included being a mother [39], a higher level of education [37], unemployment [38], and lower socioeconomic status [29,37]. Some studies found increasing child’s age [42,57] and increasing parental age [57] to be associated with decreased physical caretaker QoL and increased psychosocial caretaker QoL. In contrast, another study found increasing child’s age to be associated with lowered general parental QoL [39]. Parent-reported child’s QoL was positively associated with parents’ psychosocial and physical QoL [34,35].

Although coping plays an important role in the theoretical frameworks of caregiving of children with chronic diseases, only three studies focused on this predictor. Wiedebusch et al., 2008 found parents’ QoL to be predicted by psychosocial strains and the coping strategy ‘improving marital relationship’, jointly explaining 49% of the variance [33]. Moreover, an Iranian study found that religious coping was significantly associated with mothers’ QoL [37]. In contrast, a study with parents of children with phenylketonuria did not find general coping to be a significant predictor of parental QoL [32]. Although none of the studies investigated family functioning as a predictor of parental QoL, several studies using the instrument PedsQL™ Family Impact Module included family functioning as a subscale [31,52,53,54,55].

Although disease-specific predictors may play an important role in some diseases, the included studies show that psychosocial predictors appear to be more crucial for parental QoL. Some studies have even shown the variance explanation of psychosocial predictors to be considerably higher than disease-specific predictors [32,33,42].

### 3.7. Parental QoL by Type of Rare Disease

Due to the great heterogeneity of rare diseases in the included studies, only five diseases were investigated in more than one study. Studies reported consistent results regarding the QoL of parents of children with cystic fibrosis [39,48,59], hemophilia [33,47], osteogenesis imperfecta [30,46,52], sickle cell disease, [40,43], and phenylketonuria [32,36,38,42]. Further details of the results are given in Supplementary File S4.

## 4. Discussion

Although there is increasing research on rare diseases, parents of children with rare diseases have not been examined comprehensively. This study gives insight into the complex field of caring for a rare diseased child by providing a systematic narrative review regarding the QoL of parents of children with rare diseases relative to healthy controls and parents of children with other chronic conditions and identifies important factors associated with parental QoL, following the criteria established by the PRISMA guidelines.

Our first objective was to investigate the nature of the QoL in parents caring for children and adolescents with rare diseases. The included studies showed that affected parents had poorer QoL relative to healthy controls or norm values but similar QoL as parents of children with other chronic diseases. This especially applies to mental aspects of parental QoL and aligns with previous research on chronic diseases, showing that affected parents experience poorer QoL compared to parents of healthy children [60,61]. Unsurprisingly, the rare diseased children’s increased need for care and the resulting parental burden, limited social contacts, and family interaction was associated with a reduced QoL. Mothers had lower QoL relative to fathers, especially regarding psychosocial aspects [32,33,34,42,44]. Because the majority of studies on parental QoL have focused mainly on mothers, we were unable to determine whether this outcome was due to gender differences or whether it was associated with the role of primary caregiver. Although mothers are often more involved in the care process than their male counterparts, taking on the role of primary caregiver, the child’s rare disease impacts the entire family. Accordingly, future studies should focus on both parents’ caregiving roles and possible gender differences.

Our second objective was to identify predictors of QoL as a primary outcome of parents caring for a child with rare diseases. The studies mainly investigated clinical features and psychosocial factors as predictors of parental QoL. Important psychosocial coping factors such as stress management and family functioning were hardly examined. Although disease severity was identified as an important predictor, psychosocial factors represented more reliable predictors for parental QoL than disease-specific factors. Thus, the results of this systematic review highlight the importance of psychosocial factors, which alleviate the severity of the child’s disease as a significant stressor regarding parental QoL. The results suggest that, although disease severity and other disease-specific factors may be important predictors of lowered parental QoL, the essential predictors may be psychosocial factors. Future studies should also focus on theory-based psychosocial factors such as coping mechanisms, family functioning, and social support, which may directly impact parental QoL, but have received less attention within studies so far. The Caregiving Process and Caregiver Burden Model is particularly suited to describing the caregiving process for pediatric populations [13]. Even though the included studies investigated a wide variety of rare diseases, the impairment in parental QoL was similar across disease groups. This also applies to diseases investigated across multiple studies, all yielding similar results regarding parental QoL within specific diseases. However, a comparison of the effects of different rare diseases on parental QoL is difficult. More research on specific rare disease groups is needed in order to assess this topic comprehensively.

The results of the quantitative studies reported here and the theoretical models make it clear that interventions should follow a family-centered approach including individual sessions with the family members. Programs should focus on improving parental skills in coping with disease-specific limitations and reducing stress in the family. Furthermore, an acceptance of the impact of the respective rare disease on the parents should help alleviate the feeling of isolation. Another helpful tool may be connecting families to community support services, guiding parents in recognizing their own signs of poor mental health, and seeking help when necessary. Furthermore, affected parents should be reminded of the importance of their own psychological well-being to activate appropriate resources when needed and improve their own and their child’s well-being. Although there seems to be a great psychosocial need for parents of children with rare diseases and their families, there are hardly any interventions for this cohort. One family-centered treatment program has recently been developed in Germany and is being evaluated in a multicenter randomized controlled trial [62].

In the last two decades, several countries have established health care policies to address the needs of patients with rare diseases [24]. The results of this systematic review reflect the achievements in the implementation of these policies and provide insight into unmet health care needs. In systematically reviewing this research area, we found multiple gaps. Only a few studies have investigated the parental QoL for parents of children within one specific rare disease, a possible reason for this imbalance being the increased likeliness to investigate diseases with a relatively higher prevalence. Therefore, future research should focus not only on diseases with a higher prevalence, but also on considerably rarer, less prevalent diseases. Several of the reviewed studies showed relatively small sample sizes. Future studies should aim for appropriate sample sizes in order to achieve sufficient power. Fathers’ QoL should also be considered in more detail, as fathers and mothers have been shown to differ in their QoL. Another gap in previous research is that most studies used a cross-sectional study design, whereas only three studies investigated parental QoL at two time points [45,49,55]. Based on these study designs, the natural course of parental QoL throughout their child’s management of the disease is unknown and presents an area for future research. Furthermore, the establishment of the clinical diagnosis of the child was unclear in multiple studies. The diagnosis should be verified either by clinical evaluation, diagnostic testing, or both in future research. Within the reviewed studies, reporting could often have been improved significantly by providing information such as the comparison between responders and non-responders. Finally, the relationship between child’s age and parental QoL should be examined more closely in longitudinal studies to clarify whether the parents’ burden decreases as their child grows older.

### Study Limitations

While the findings of this review provide valuable information regarding the research field of rare diseases, there are limitations to consider. First, the systematic review contained only articles published in English and German. Hence, additional studies might have been overlooked. Second, most of the studies relied on population-based norms to describe parental QoL. Only three studies [31,33,57] directly compared the QoL of parents of children with a rare disease with parents of children with other chronic conditions. This represents an area for future research and would help identify the impact on parental QoL beyond the challenges and social limitations associated with parenting children with chronic diseases. Third, few studies have explicitly linked their research to a theoretical framework. The use of a theoretical model should be a crucial component of high-quality research, guiding hypotheses and methods. Finally, the wide range of study quality and QoL instruments as well as the limited number of studies dealing with specific rare diseases should be considered when interpreting the results. Due to the heterogeneity of rare diseases, the different QoL instruments used in these studies and their study designs, a meta-analytic evaluation was omitted.

## 5. Conclusions

Despite these limitations, the results of this systematic review indicate that parents of children with rare diseases experience an impairment in their QoL in comparison to norm values and parents of children with other chronically ill children. Psychosocial parental QoL seems to be primarily affected. The results of this review can be used to guide further studies in the field of rare diseases and thus fill relevant research gaps. We want to emphasize the need for validated instruments within a theory-driven approach for future research.

## Figures and Tables

**Table 1:** Search strategies.

	Ovid Databases (APA PsycArticles, APA PsycInfo, MEDLINE, PSYNDEXplus)	PubMed
1	((rare or orphan or genetic or chronic) adj (disease * or diagnos * or condition * or disorder * or illness *)).mp.	(“rare disease *”[Text Word] OR “orphan disease *”[TW] OR “genetic disease *”[TW] OR “chronic disease *”[TW] OR “rare diagnos *”[TW] OR “genetic diagnos *”[TW] OR “chronic diagnos *”[TW] OR “rare condition *”[TW] OR “orphan condition *”[TW] OR “genetic condition *”[TW] OR “chronic condition *”[TW] OR “rare disorder *”[TW] OR “orphan disorder *”[TW] OR “genetic disorder *”[TW] OR “chronic disorder *”[TW] OR “rare illness*”[TW] OR “orphan illness *”[TW] OR “genetic illness *”[TW] OR “chronic illness *”[TW])
2	(child * or paediatric or pediatric or daughter or son).mp.	(“child *”[TW] OR “paediatric”[TW] OR “pediatric”[TW] OR “daughter”[TW] OR “son”[TW])
3	(caregiv * or parent * or mother * or father * or famil * or carer * or foster * or guardian *).mp.	(“caregiv *”[TW] or “parent *”[TW] or “mother *”[TW] or “father *”[TW] or “famil *”[TW] or “carer *”[TW] or “foster *”[TW] or “guardian *”[TW])
4	(quality of life or QoL or HRQoL).mp.	(“HRQoL”[TW] or “quality of life”[TW] or “QoL”[TW])
5	1 and 2 and 3 and 4	1 and 2 and 3 and 4
6	limit 5 to year = “2000–2019”	(2000/01/01[pdat]:2019/12/31[pdat])
7	limit 6 to original articles	(“journal article”[PT])
8	remove duplicates from 7	

Note. QoL = Quality of Life, HRQoL = Health-Related Quality of Life. Source: Own elaboration.

**Table 2:** List of rare diseases by clinical category.

Clinical Category	Disease (Abbreviation)	Prevalence	Number of Studies
Chromosomal disorders	Down syndrome (Trisonomy 21)	1–5/10,000	1
	Potocki–Lupski syndrome (Trisonomy 17p11.2)	1/25,000	1
	Prader-Willi syndrome (PWS)	1–9/100,000	1
	Wiskott–Aldrich syndrome (WAS)	1–9/1,000,000	1
Congenital diseases	Anorectal malformation (ARM)	n/a	1
	Esophageal atresia (EA)	1–5/10,000	1
	Hirschsprung disease (HD)	1–5/10,000	1
	Spina bifida	1–5/10,000	1
Hematologic and oncologic diseases	Hemophilia	1–9/100,000	2
	Pediatric leukemia	n/a	1
	Sickle cell disease	1–5/10,000	2
	Tuberous sclerosis complex	1–5/10,000	1
	X-Linked Thrombocytopenia	n/a	1
Inflammatory diseases	Cystic fibrosis (CF)	1–9/100,000	4
	Juvenile idiopathic arthritis (JIA)	45/100,000	1
Metabolic disorders	Galactosemia	n/a	1
	Phenylketonuria (PKU)	1–5/10,000	4
	Methylmalonic acidemia	1–9/100,000	1
	Mucopolysaccharidosis type II (MPS2)	1–9/1,000,000	1
	Tyrosinemia type 1 (HT1)	<1/100,000	1
Musculoskeletal diseases	Achondroplasia	1–9/100,000	1
	Osteogenesis imperfecta (OI)	1–5/10,000	3
Neuromuscular and neurologic disorders	CDKL5 deficiency disorder	1/40,000–1/60,000	1
	Duchenne muscular dystrophy (DMD)	1–9/100,000	1
	Pediatric multiple sclerosis (MS)	n/a	1
	Rett syndrome	1–9/100,000	1

Note: Prevalence of rare disease is extracted from www.orpha.net, accessed on 5 January 2021. n/a: not applicable. Source: Own elaboration based on the data obtained in the study.

**Table 3:** List of quality of life instruments.

Abbreviation	Name	Objective	Number of Studies
BCFQOL	Beach Center Family Quality of Life	Generic QoL	1
CarerQol-7D	Care-related Quality of Life instrument	Generic QoL	1
CQOLCF	Caregiver Quality of Life Cystic Fibrosis	Disease-specific QoL	3
CQOLC	Caregiver Quality of Life Index-Cancer	Disease-specific QoL	1
PedsQL™ Family Impact Module	Pediatric Quality of Life Inventory™ Family Impact Module	Generic HRQoL	7
SF-8	Short Form Health Survey-8 Items	Generic QoL	2
SF-12	Short Form Health Survey-12 Items	Generic QoL	3
SF-36	Short Form Health Survey-36 Items	Generic QoL	3
TAAQoL	TNO-AZL Questionnaire for Adult’s Health-related Quality of Life	Generic HRQoL	4
TYR-QOL	Tyrosinemia Quality of Life Caregiver Questionnaire	Disease-specific QoL	1
ULQIE	Ulm Quality of Life Inventory for Parents of Chronically Ill Children	Generic QoL	2
WHOQOL-BREF-TR	World Health Organization Quality of Life Questionnaire-Short Form	Generic QoL	4

Note. Source: Own elaboration based on the data obtained in the study.

## Data Availability

The data presented in this study are available in Supplementary File S4.

## Supplementary Materials

The following are available online at https://www.mdpi.com/article/10.3390/ijerph18094993/s1, Supplementary File S1: PRISMA 2009 Checklist, Supplementary File S2: Quality assessment of included cross-sectional studies on parental QoL, Supplementary File S3: Quality assessment of included cohort studies on parental QoL, Supplementary File S4: Summary of studies on parental QoL.
