# Supplementary material for: Being the Pillar for Children with Rare Diseases—A Systematic Review on Parental Quality of Life
Source: Int J Environ Res Public Health. 2021 May 8;18(9):4993. doi: 10.3390/ijerph18094993 (PMC8125857; doi:10.3390/ijerph18094993)
Supplement: Supplementary file 1 [file ijerph-18-04993-s001.zip › Supplementary File S2.pdf]

Supplementary File S2. Quality assessment of included cross-sectional studies on parental QoL.

Author	Selection				Comparability	Outcome		Total
	A	B	C	D	E	F	G	
Witt et al. (2019)	*	*		*		*	*	5
Mori et al. (2017)	*	*		*	**	*	*	7
Boling (2005)	*	*		*		*		4
Driscoll et al. (2009)	*	*		*	**	*	*	7
Fitzgerald et al. (2018)	*	*		*	**	*	*	7
Witt et al. (2018)	*	*	*	*		*	*	6
Lindvall et al. (2014)	*	*		*	**	*	*	7
Wiedebusch et al. (2008)	*			*	**	*	*	6
Haverman et al. (2014)	*	*	*	*	**	*	*	8
Khanjari et al. (2018)	*	*		*	**	*		6
Splinter et al. (2016)	*	*		*		*		4
Needham et al. (2014)	*	*		*		*	*	5
O'Mahony et al. (2019)	*	*	*	*		*	*	6
Lazow et al. (2019)	*		*	*		*		4
Szczepaniak-Kubat et al. (2012)	*			*		*	*	4
Vanz et al. (2015)	*			*	**	*	*	6
Fidika et al. (2013)	*	*		*	**	*	*	7
Irannejad et al. (2018)	*	*		*		*		4
Mahmoudi-Gharaei et al. (2011)	*	*		*	**	*	*	7
Carter et al. (2013)	*			*		*	*	4
Mazaheri et al. (2013)	*			*		*	*	4
Van Den Tweel et al. (2008)	*	*	*	*	**	*	*	8
Rentz et al. (2015)	*	*		*		*	*	5
Campbell et al. (2018)	*			*		*	*	4
Hatzmann et al. (2008)	*	*	*	*		*	*	8
ten Hoedt et al. (2011)	*	*		*	**	*	*	7
Witvliet et al. (2014)	*	*		*		*		4
Shah et al. (2019)	*	*		*		*	*	5

Note. A study can be awarded a maximum of one star for each numbered item within the Selection and Outcome categories. A maximum of two points can be given for Comparability. Quality assessment scores were assessed with the modified Newcastle-Ottawa Scale for cross-sectional studies (very good = 7-8, good = 5-6, satisfactory = 4, unsatisfactory = 0-3). (A) Representativeness of the sample: Truly representative of the average in the target population or somewhat representative of the average in the target population (one point); no description (no point), (B) Sample size justified: Justified and satisfactory (one point); not justified (no point), (C) Non-respondents: Comparability between respondents and non-respondents characteristics is established, and the response rate is satisfactory (one point); the response rate is unsatisfactory, or the comparability between respondents and non-respondents is unsatisfactory or no description of the response rate or the characteristics of the responders and the non-responders (no point), (D) Ascertainment of exposure: Assessment using validated measurement tool (one point); Non-validated measurement tool or no description of the measurement tool (no point), (E) The subjects in different outcome groups are comparable, based on the study design or analysis. Confounding factors are controlled: The study controls for the most important factors (one point); The study controls for any additional factors (one point), (F) Assessment of the outcome: self-report (one point); no description (no point),(G) Statistical test: The statistical test used to analyze the data clearly described, appropriate and measures of the association presented including confidence intervals and probability level (one point); Statistical test not appropriate, not described or incomplete (no point). Source: Own elaboration based on the data obtained in the study.
