# Supplementary material for: Being the Pillar for Children with Rare Diseases—A Systematic Review on Parental Quality of Life
Source: Int J Environ Res Public Health. 2021 May 8;18(9):4993. doi: 10.3390/ijerph18094993 (PMC8125857; doi:10.3390/ijerph18094993)
Supplement: Supplementary file 1 [file ijerph-18-04993-s001.zip › Supplementary File S3.pdf]

Supplementary File S3. Quality assessment of included cohort studies on parental QoL.

Author	Selection				Comparability	Outcome			Total
	A	B	C	D	E	F	G	H	
Suthoff et al. (2019)	*		*	*		*	*	*	6
Killian et al, (2016)	*		*	*	**	*	*		7
Witvliet et al. (2015)	*		*	*		*	*		4

Note. A study can be awarded a maximum of one star for each numbered item within the Selection and Outcome categories. A maximum of two points can be given for Comparability. Quality assessment scores were assessed with the Newcastle-Ottawa Scale for cohort studies (very good = 8-9, good = 6-7, satisfactory = 4-5, unsatisfactory = 0-3). (A) Representativeness of the sample: Truly representative of the average in the target population or somewhat representative of the average in the target population (one point); no description (no point), (B) Selection of the non-exposed cohort: drawn from the same community as the exposed cohort (one point); drawn from a different source or no description of the derivation of the non-exposed cohort, (C) Ascertainment of exposure: secure record (one point); written self-report or no description (no point), (D) Demonstration that outcome of interest was not present at the start of the study: yes (one point), no (no point), (E) Comparability of cohorts on the basis of the design or analysis: The subjects in different outcome groups are comparable, based on the study design or analysis. Confounding factors are controlled (two points); results are not adjusted for all relevant confounders or risk factors or information not provided (no points), (F) Assessment of outcome: self-report (one point); No description (no point), (G) Follow-up long enough for outcomes to occur: yes (one point); no (no point), (H) Adequacy of follow-up of cohorts: subjects lost to follow-up unlikely to introduce bias or inadequate numbers but the description provided of those lost (one point); Inadequate follow-up rate or no description of those lost (no points). Source: Own elaboration based on the data obtained in the study.
