# Supplementary material for: Being the Pillar for Children with Rare Diseases—A Systematic Review on Parental Quality of Life
Source: Int J Environ Res Public Health. 2021 May 8;18(9):4993. doi: 10.3390/ijerph18094993 (PMC8125857; doi:10.3390/ijerph18094993)
Supplement: Supplementary file 1 [file ijerph-18-04993-s001.zip › Supplementary File S4.pdf]

Supplementary File S4. Summary of studies on parental QoL.

Rare disease	References	Country	Study population	QoL Scale	Selected findings
Achondroplasia	Witt et al. (2019)[CS]	Germany	56 female and 17 male caregivers of children (5-14 yrs) with achondroplasia	SF-8	Parents reported significantly lower QoL in the mental subscale compared to norm data of the German reference population, whereas this could not be found for the physical subscale.<br>In both QoL subscales, the higher the parental QoL, the higher the parent-reported children's HRQoL.
CDKL5 deficiency disorder	Mori et al. (2017)[CS]	Australia	141 female and 15 male caregivers of children (1-13 yrs) with CDKL5 deficiency disorder	SF-12<br>BCFQOL	Mothers' QoL was significantly lower in the mental subscale in comparison to US female norms; in contrast, mothers' physical QoL subscale was significantly higher.<br>Mental QoL was adversely affected by experiencing financial hardship and the severity of the child's sleep disturbances.<br>Family QoL was generally rated as satisfactory.
Cystic fibrosis	Boling (2005)[CS]	USA	81 female and 19 male caregivers of children with cystic fibrosis	CQOLCF	As the child's disease severity increased, the caregiver's QoL decreased, although not significantly, when adjusted for age.<br>As the days of the child's hospitalization increased, the caregiver's QoL significantly increased when adjusted for age.
Cystic fibrosis	Driscoll et al. (2009)[CS]	USA	100 female and 22 male caregivers of children (0-17 yrs) with cystic fibrosis	CQOLCF	Caregiver QoL was associated with both depressive and anxious symptoms, whereas fewer symptoms were associated with better QoL.<br>Disease severity was significantly associated with mothers', but not fathers' QoL.
Cystic fibrosis	Fitzgerald et al. (2018)[CS]	Ireland	189 female and 137 male caregivers of children with cystic fibrosis	CarerQol-7D	Increased child age, being a mother, and an infection with Pseudomonas aeruginosa were significantly associated with lowered parental QoL.
Cystic fibrosis	Suthoff et al. (2019)[L]	USA	74 female and 14 male caregivers of children (2-18 yrs) with cystic fibrosis	SF-12<br>CQOLCF	Mental subscales (SF-12) of QoL were significantly lowered during pulmonary exacerbation-related hospitalization compared to the time point after a "well state" of the child. No significant difference between those time points was found for the physical QoL subscale.<br>No significant difference between both time points was found for the disease-specific QoL instrument (CQOLCF).

Esophageal atresia	Witt et al. (2018)[CS]	Germany	47 female and 40 male caregivers of children (2-17 yrs) with esophageal atresia	SF-8	Affected parents reported significantly lower scores in the mental but not physical subscale of QoL compared with the general population. Mothers in comparison to fathers reported significantly lower QoL in mental subscales. Mental and physical QoL of the parents were significantly associated with the parent-reported QoL of the child.
Hemophilia	Lindvall et al. (2014)[CS]	Sweden	42 female and 34 male caregivers of children (1-18 yrs) with hemophilia	SF-36	Parents reported significantly decreased QoL in all subscales, except for physical subscales pain and general health, compared to parents of healthy children. No significant difference was found between parents of children with and without inhibitors.
Hemophilia	Wiedebusch et al. (2008)[CS]	Germany	32 female and 23 male caregivers of children (1-20 yrs) with hemophilia	ULQIE	Parents reported significantly higher QoL than parents of children with juvenile idiopathic arthritis and type I diabetes. No significant difference was found between mothers' and fathers' QoL.<br>Predictors of QoL showed that parents who reported a better marital relationship and fewer emotional strains and worries had a higher QoL.
Juvenile idiopathic arthritis	Haverman et al. (2014)[CS]	Netherlands	147 female and 8 male caregivers of children (0-18 yrs) with juvenile idiopathic arthritis	TAAQoL	Parents reported significantly lower HRQoL in the subscale fine motor functioning and significantly higher HRQoL in the subscale social functioning in comparison to the Dutch normative sample.<br>Parents of children with active juvenile idiopathic arthritis showed worse HRQOL regarding the subscales daily activities, cognitive functioning, and depressive emotions compared to parents of children without active juvenile idiopathic arthritis.
Pediatric leukemia	Khanjari et al. (2018)[CS]	Iran	200 mothers of children (1-15 yrs) with pediatric leukemia	CQOLC	Protective factors like education level, income, and occupation had a significant statistical relationship with general QoL in affected mothers.
Methylmalonic acidemia	Splinter et al. (2016)[CS]	USA	35 caregivers of children (2-18 yrs) with methylmalonic acidemia	PedsQL™ Family Impact Module	Parents reported lower general QoL in comparison to norm data of parents with chronic health conditions living in a long-term care facility. QoL was comparable to parents of children with chronic conditions living at home.<br>No significant difference was found between parents of children with and without liver transplantation.

Mucopolysaccharidosis type II	Needham et al. (2014)[CS]	USA	73 parents of children (2->21 yrs) with Mucopolysaccharidosis type II	PedsQL™ Family Impact Module	Parents experienced lower QoL in comparison to norm data of parents of children with chronic health conditions living in a long-term care facility, whereas no differences compared to norm data of parents of children with chronic conditions living at home were found.<br>Findings showed that severity of illness was negatively, and time on enzyme replacement therapy was positively associated with parental QoL.
Pediatric multiple sclerosis	O'Mahony et al. (2019)[CS]	Canada	58 parents of families with Multiple sclerosis	PedsQL™ Family Impact Module	Parents caring for children with multiple sclerosis reported significantly lower QoL in all subscales compared to parents of children with monophasic (non-chronic) acquired demyelinating syndrome.
Osteogenesis imperfecta	Lazow et al. (2019)[CS]	USA	22 female and 8 male caregivers of children with osteogenesis imperfecta	PedsQL™ Family Impact Module	The child's pain ratings and physical functioning were significantly associated with lowered parental QoL.
Osteogenesis imperfecta	Szczepaniak-Kubat et al. (2012)[CS]	Poland	25 female and 24 male caregivers of children with osteogenesis imperfecta	WHOQOL-BREF-TR	Affected parents showed no significant difference in QoL in comparison to norm data of the general population.<br>Parents of children with severe osteogenesis imperfecta showed significantly lower QoL in the environmental subscale in comparison to parents of children with mild osteogenesis imperfecta.
Osteogenesis imperfecta	Vanz et al. (2015)[CS]	Brazil	18 female and 6 male caregivers of children with osteogenesis imperfecta	WHOQOL-BREF-TR	Affected parents reported significantly lower QoL in the subscales physical, psychological, and environmental in comparison to the Brazilian norm data.<br>Socioeconomic status and number of fractures sustained by the care recipient were not significantly associated with parental QoL.
Phenylketonuria	Fidika et al. (2013)[CS]	Germany	76 female and 13 male caregivers of children (1-19 yrs) with phenylketonuria	ULQIE	Family stress and perceived social support were identified as significant predictors for parental QoL.<br>The results indicated no significant differences between mothers and fathers for all QoL subscales.
Phenylketonuria	Irannejad et al. (2018)[CS]	Iran	124 caregivers of children (<18 yrs) with phenylketonuria	SF-36	Age of child and perceived stress were significantly associated with parental QoL.

Phenylketonuria	Mahmoudi-Gharaei et al. (2011)[CS]	Iran	29 female and 20 male caregivers of children with phenylketonuria	WHOQOL-BREF-TR	Significant predictors of parental QoL were caregiver's occupation, stress, anxiety, and depression.
Potocki-Lupski syndrome	Carter et al. (2013)[CS]	USA	25 female and 4 male caregivers of 29 children (0-17 yrs) with Potocki-Lupski syndrome	PedsQL™ Family Impact Module	Lower QoL scores were found in parents of children with the presence of feeding difficulties in comparison to children absent of feeding difficulties. Higher QoL scores were found in parents of children with the presence of cardiovascular defects compared to no defects.
Prader-Willi syndrome	Mazaheri et al. (2013)[CS]	USA	12 mothers of children (1-27 yrs) with Prader-Willi syndrome	PedsQL™ Family Impact Module	Mothers showed significantly lower perceived QoL compared with norm data of parents of children with complex health conditions in long-term care convalescent hospital and mental QoL subscales compared with norm data of parents of children with complex health conditions that live at home.
Rett syndrome	Killian et al, (2016)[L]	USA	220 mothers of children with Rett syndrome	SF-36	Mothers' physical and mental QoL was not significantly different in five-year follow-up compared to baseline assessment.<br>Increasing child age, increasing parental age, and higher disease severity were associated with decreased physical QoL and increased mental QoL.<br>Child psychosocial QoL subscales were significantly associated with parental mental QoL.
Sickle cell disease	Van Den Tweel et al. (2008)[CS]	Netherlands	54 mothers of children (1-17 yrs) with sickle cell disease	TAAQoL	Mothers reported significantly lower HRQoL on all subscales compared to the Dutch norm population.<br>Mothers reported significantly lower HRQoL on the subscale's depressive moods, daily activities, and vitality in comparison to a healthy control group matched for SES and ethnicity.
Tuberous sclerosis complex	Rentz et al. (2015)[CS]	USA	149 female and 27 male caregivers of children with tuberous sclerosis complex	SF-12	Caregivers showed significantly lower QoL in all subscales compared to norms of US healthy adult population.<br>Caregivers of pediatric patients reported significantly higher QoL in four of eight subscales and the mental component summary in comparison to caregivers of adult patients.
Tyrosinemia type 1	Campbell et al. (2018)[CS]	USA	20 female and 5 male caregivers of children with Tyrosinemia type 1	TYR-QOL	The emotional, practical, social, and overall impact of QOL were higher for parents of children with tyrosinemia type 1 compared to parents of children with mild Phenylketonuria, but not classic Phenylketonuria.

Multiple rare diseases<br>Down syndrome, Duchenne muscular dystrophy, Sickle cell disease, Spina bifida	Hatzmann et al. (2008)[CS]	Netherlands	170 female and 31 male caregivers of children with different rare diseases	TAAQoL	Parents of children with sickle cell disease showed significantly lower HRQoL in almost all subscales except fine motor functioning compared to parents of healthy children. Parents of children with Duchenne muscular dystrophy showed significant impairment in social functioning, sexuality, vitality, and positive and depressed emotions. Parents of children with down syndrome showed significant impairment of cognitive and social functioning, daily activities, and vitality. Parents of children with spina bifida showed significant impairment in fine motor function, daily activities, and vitality.
Multiple rare diseases<br>Phenylketonuria and Galactosemia	ten Hoedt et al. (2011)[CS]	Netherlands	66 female and 50 male caregivers of children (1-19 yrs) with phenylketonuria and 42 female and 27 caregivers of children (1-19 yrs) with galactosemia	TAAQoL	Parents of children with phenylketonuria or galactosemia reported a HRQoL comparable to parents of healthy children and a significantly better HRQoL than parents of children with other metabolic disorders.<br>Significant predictors for mental HRQoL in parents of phenylketonuria were higher age of the child, emotional support, and loss of friendship, whereas, in parents of children with galactosemia, only emotional support was found to be a significant predictor for mental HRQoL.<br>Mothers experienced lower mental HRQoL than fathers, although it did not reach significance.
Multiple rare diseases<br>Anorectal Malformation and Hirschsprung Disease	Witvliet et al. (2014, 2015)[CS,L]	Netherlands	44 female and 42 male caregivers of children (0-13 yrs) with anorectal malformation or Hirschsprung disease	WHOQOL-BREF-TR	Mothers reported significantly lower QoL on the psychological subscale compared to fathers.<br>Dividing the group into parents of newborns and older children showed no significant difference in psychological QoL between mothers and fathers.<br>Mothers scored lower on the social and environmental subscale and higher on the physical, psychological and overall domain after one year. Fathers scored higher on the psychological and overall domain after one year. There was no significant difference between mothers and fathers.
Multiple rare diseases<br>Wiskott-Aldrich Syndrome & X-Linked Thrombocytopenia	Shah et al. (2019)[CS]	USA	47 caregivers of patients with Wiskott Aldrich Syndrome or X-Linked Thrombocytopenia	PedsQL™ Family Impact Module	Parents showed lower scores compared with norm data of healthy parents in the subscales emotional function, social function, communication, and worry.

Note. CS = cross-sectional, L = longitudinal. QoL = Quality of Life, HRQoL = Health Related Quality of Life. Source: Own elaboration based on the data obtained in the study.
